# New Tmc1 Deafness Mutations Impact Mechanotransduction in Auditory Hair Cells

**DOI:** 10.1523/JNEUROSCI.2537-20.2021

**Published:** 2021-05-19

**Authors:** Maryline Beurg, Lisa A. Schimmenti, Alaa Koleilat, Sami S. Amr, Andrea Oza, Amanda J. Barlow, Angela Ballesteros, Robert Fettiplace

**Affiliations:** ^1^Department of Neuroscience, University of Wisconsin School of Medicine and Public Health, Madison, Wisconsin 53706; ^2^Departments of Clinical Genomics, Otorhinolaryngology, Head and Neck Surgery and Biochemistry and Molecular Biology, Mayo Clinic School of Medicine, Rochester, Minnesota 55902; ^3^Department of Laboratory Medicine and Pathology, Mayo Clinic, Rochester, Minnesota 55902; ^4^Laboratory for Molecular Medicine, Partners HealthCare Personalized Medicine, Cambridge, Massachusetts 02139; ^5^Partners HealthCare Personalized Medicine, Laboratory for Molecular Medicine, Cambridge, Massachusetts 02139; ^6^Molecular Physiology and Biophysics section, National Institute of Neurological Disorders and Stroke-National Institutes of Health, Bethesda, Maryland 20892; ^7^Department of Pathology, Brigham and Women's Hospital, Harvard Medical School, Boston, Massachusetts 02115

**Keywords:** cochlea, deafness, Hair cell, mechanotransduction channel, TMC1

## Abstract

Transmembrane channel-like protein isoform 1 (TMC1) is a major component of the mechano-electrical transducer (MET) channel in cochlear hair cells and is subject to numerous mutations causing deafness. We report a new dominant human deafness mutation, *TMC1* p.T422K, and have characterized the homologous mouse mutant, *Tmc1* p.T416K, which caused deafness and outer hair cell (OHC) loss by the fourth postnatal week. MET channels showed decreased Ca^2+^ permeability and resting open probability, but no change in single-channel conductance or expression. Three adjacent deafness mutations are *TMC1* p.L416R, p.G417R, and p.M418K, the last homologous to the mouse *Beethoven* that exhibits similar channel effects. All substitute a positive for a neutral residue, which could produce charge screening in the channel pore or influence binding of an accessory subunit. Channel properties were compared in mice of both sexes between dominant (*Tmc1* p.T416K, *Tmc1* p.D569N) and recessive (*Tmc1* p.W554L, *Tmc1* p.D528N) mutations of residues near the putative pore of the channel. *Tmc1* p.W554L and p.D569N exhibit reduced maximum current with no effect on single-channel conductance, implying a smaller number of channels transported to the stereociliary tips; this may stem from impaired TMC1 binding to LHFPL5. *Tmc1* p.D528N, located in the pore's narrowest region, uniquely caused large reductions in MET channel conductance and block by dihydrostreptomycin (DHS). For *Tmc1* p.T416K and *Tmc1* p.D528N, transduction loss occurred between P15 and P20. We propose two mechanisms linking channel mutations and deafness: decreased Ca^2+^ permeability, common to all mutants, and decreased resting open probability in low Ca^2+^, confined to dominant mutations.

**SIGNIFICANCE STATEMENT** Transmembrane channel-like protein isoform 1 (TMC1) is thought to be a major component of the mechanotransducer channel in auditory hair cells, but the protein organization and channel structure are still uncertain. We made four mouse lines harboring *Tmc1* point mutations that alter channel properties, causing hair cell degeneration and deafness. These include a mouse homolog of a new human deafness mutation pT416K that decreased channel Ca^2+^ permeability by introducing a positively-charged amino acid in the putative pore. All mutations are consistent with the channel structure predicted from modeling, but only one, p.D528N near the external face of the pore, substantially reduced channel conductance and Ca^2+^ permeability and virtually abolished block by dihydrostreptomycin (DHS), strongly endorsing its siting within the pore.

## Introduction

Sound detection occurs in the cochlea where evoked mechanical stimuli are translated into electrical signals by the gating of mechano-electrical transducer (MET) channels in the stereocilia of sensory hair cells (Fettiplace, 2017). A bundle of 50–100 stereocilia arranged in three or four rows of increasing height projects from the endolymphatic surface of each hair cell. The MET channels, located at the lower end of each tip link, are activated by force delivered via interciliary tip links (Pickles et al., 1984; Assad et al., 1991; Beurg et al., 2009). The MET channel is thought to be formed principally by the transmembrane channel-like protein isoform 1 (TMC1) protein (Kawashima et al., 2011; Kim and Fettiplace, 2013; Pan et al., 2018), with additional proteins serving as accessory subunits; these include LHFPL5 (Xiong et al., 2012), TMIE (Zhao et al., 2014a; Pacentine and Nicolson, 2019; Cunningham et al., 2020), and CIB2 (Giese et al., 2017). There are at least 35 reported point mutations (pathogenic variants) of the *TMC1* gene causing human deafness (Yue et al., 2019), underscoring the key role of TMC1 in sound transduction. These mutations are divided into two categories, those referred to as DFNA36 that are autosomal dominant, and those labeled DFNB7/11 that are autosomal recessive (Kurima et al., 2002). A previously described dominant mouse mutation, *Tmc1* p.M412K (*Beethoven*), leads to early hearing loss and hair cell degeneration (Vreugde et al., 2002). The homologous human deafness mutation is *TMC1* p.M418K (Zhao et al., 2014b; Wang et al., 2018), the mutation site is adjacent to two other missense mutations causing deafness: *TMC1* p.L416R (Chen et al., 2015) and *TMC1* p.G417R (Yang et al., 2010). Here we report a fourth deafness mutation in the same region, *TMC1* p.T422K and we generate and characterize the homologous mouse mutation *Tmc1* p.T416K.

The phenotypic changes in MET channel properties in *Tmc1* p.M412K have been well described (Vreugde et al., 2002; Marcotti et al., 2006; Pan et al., 2013; Beurg et al., 2015a; Corns et al., 2016) but the mechanism leading to hair cell degeneration and death is unknown. The decrease in MET channel Ca^2+^ permeability in *Tmc1* p.M412K is semi-dominant, the heterozygous possessing a permeability intermediate between the wild type and homozygote (Corns et al., 2016; Beurg et al., 2019). Localization of the mutation site has been gleaned from recent modeling of the TMC1 protein structure based on its relationship with TMEM16 chloride channels and lipid scramblases, which have 10 transmembrane (TM) domains (Ballesteros et al., 2018; Pan et al., 2018). This modeling suggests that the pore cavity is formed by TM domains TM4 to TM7, and maps the region containing the string of four mutants to TM4. Several sites crucial for MET channel performance have been identified by adenovirus-mediated expression of TMC1 containing cysteine substitutions in neonatal mice, and then treating cultured cochleas with cysteine modification reagents (Pan et al., 2018). We have extended that work using the CRISPR/Cas9 technology to generate mice with missense mutations in TMC1, which enabled us to characterize the deafness phenotype, whether dominant or recessive, and possible mechanism relating to hearing loss in the unaltered endogenous tissue. We also examined the *stitch* mutant mouse, *Tmc1* p.W554L (Manji et al., 2012), which is located at the intracellular loop joining TM6 and TM7 in the TMC1 model. We compare its properties with those of other deafness mutants *Tmc1* p.D569N (Beurg et al., 2019) and *Tmc1* p.D528N. These experiments together address the assumptions that TMC1 is the major component of the MET channel and that the region comprising TM4 to TM7 at least partly dictates ionic performance.

## Materials and Methods

### 

#### Human subjects

The Mayo Clinic Institutional Review Board (IRB) provided approval for this study.

#### Mouse mutants

*Tmc1* p.D569N and *Tmc1* p.W554L mutant mice were made by Applied StemCell Inc (Beurg et al., 2019) and *Tmc1* p.D528N and *Tmc1* p.T416K mice were made by Horizon Sage Labs Inc, all using CRISPR/Cas9 technology. For all four genotypes, mutations were verified by 500 base pair sequencing around the mutation site, and mice were subsequently bred for five generations, after which any off-target effects should have been eliminated. *Tmc1* p.M412K *Beethoven* mice were a gift of Walter Marcotti (Sheffield University, United Kingdom) and Karen Steel (Kings College London, United Kingdom). All *Tmc1* mutants for which hair cell transduction was characterized were performed on a *Tmc2*−/− background to avoid complications because of different channel properties of TMC2 (Kim et al., 2013; Kim and Fettiplace, 2013). *Tmc2*−/− mice (B6.129S5-*Tmc2^tm1Lex^*/Mmucd) were obtained from the Mutant Mouse Regional Resource Center (University of California, Davis, CA). Neonatal mice were killed by decapitation according to the animal protocol approved by the Institutional Animal Care and Use Committee at the University of Wisconsin-Madison. For all strains, a mixture of male and female mice was used and no gender-specific effects were noted. Acoustic brainstem responses (ABRs) and distortion product otoacoustic emissions (DPOAEs) were measured on postnatal day (P)30 and P60 mutants and P30 wild-type mice, anesthetized with 10 mg/ml ketamine plus 1 mg/ml xylazine and using Tucker-Davis Technology Auditory Workstation. Preyer (pinna twitch) reflexes were evoked using an Oxefly dog-training clicker to detect hearing in P15–P21 mice that were too young for ABR measurements. The clicker was place ∼10 cm from the back of the head.

#### Hair cell survival

To document hair cell survival in mutants, isolated cochleas were fixed in 4% paraformaldehyde for 1 h at room temperature, processed (Beurg et al., 2019) and immunolabeled with rabbit monoclonal anti-calbindinD28k (Swant, 1:500, 2 h), and Alexa Fluor 568 phalloidin. Mounted preparations were viewed with a 60× Plan apochromat (NA = 1.4) on a Nikon A1 laser-scanning confocal microscope, and fluorescence intensity was quantified with ImageJ. Two methods were used to assay the number of functional MET channels in mutants: immunolabeling of TMC1 (Beurg et al., 2015b) and hair cell accumulation of the permeant FM1-43 (Gale et al., 2001). For TMC1 immunolabeling, cochleas of P6 mice were fixed in 4% paraformaldehyde for 30 min followed by permeabilization in 0.5% Triton for 30 min at room temperature, then incubated in 10 mm Na citrate, pH 6.0, 75°C for 30 min. Fixed cochleas were subsequently immersed in 10% normal goat serum for 1 h at room temperature and incubated overnight at 4°C with the primary anti-TMC1 affinity-purified antibody (HPA 044166, made against a 39-residue N-terminal human TMC1 sequence; Sigma-Aldrich) at 1:50 dilution, followed by anti-rabbit Alexa Fluor 488 secondary antibody. Mounted preparations were viewed with a 60× Plan apochromat (NA = 1.4) on a Nikon A1 laser-scanning confocal microscope, and fluorescence intensity was quantified with ImageJ. Each immunolabeling run was performed on three cochleas from different animals, and the process was repeated two times, so six animals in total were used for each labeling procedure.

FM1-43 staining was performed on apical turns of unfixed cochlear explants of P15, P20, and P30 mice using labeling procedures described previously (Vélez-Ortega et al., 2017; Krey et al., 2020). Animals were anesthetized with isoflurane then decapitated according to the animal protocol approved by the Institutional Animal Care and Use Committee at the University of Wisconsin-Madison. A piece of temporal bone was excised and held in a thin slice of SILASTIC tubing (∼3 mm in diameter) glued to a coverslip (Mahendrasingam et al., 2010). The apical turn of the cochlea was exposed and opened, Reissner's membrane removed and the cochlear apex perfused with 0.1 mg/ml protease (type XXIV, Sigma-Aldrich) for 2 min to loosen the tectorial membrane but not to remove it. It was then incubated for 2 min in 0.1 mm tubocurarine [to block remaining MET channels, sometimes supplemented with 1 mm dihydrostreptomycin (DHS)] followed by 30 s in ice-cold HBSS supplemented with 6 μm FM1-43 (ThermoFisher) in the presence or absence of 0.1 mm tubocurarine, then washed in HBSS. The FM1-43 solution was ice cold to inhibit uptake by a metabolically sensitive endocytic mechanism. The preparation was observed on a Leica DMLFS top-focusing microscope with a 40× water-immersion objective (NA = 0.8) and imaged with an Orca camera (Hamamatsu Corporation). The mean fluorescence intensity was quantified with ImageJ in a region of interest extending to the edge of the cell, below the cuticular plate, about half-way down the OHCs and in the crook of the IHC soma where it bends toward the modiolus. The focal plane was often different for the two types of hair cell. The camera gain setting was kept constant across the different manipulations: genotypes with and without channel blocker.

#### Hair cell recording and stimulation

MET currents were recorded from inner hair cells (IHCs) and outer hair cells (OHCs) in isolated organs of Corti of mice between P2 and P8 as previously documented (Kim et al., 2013; Beurg et al., 2019). Preparations were mounted on the stage of a Leica DMLFS top-focusing microscope and viewed with 40× (NA= 0.8) objective and a 2× optivar. Most recordings were from the apical (low frequency) turn, ∼70% of the distance along the cochlea from the stapes. The recording chamber was perfused with saline: 152 mm NaCl, 6 mm KCl, 1.5 mm CaCl_2_, 2 mm Na-pyruvate, 8 mm D-glucose, and 10 mm Na-HEPES, pH 7.4. In some experiments, saline of reduced (0.04 mm) CaCl_2_ was perfused over the preparation to replace the normal (1.5 mm) CaCl_2_. In this type of experiment, the fluid jet initially contained saline with normal Ca^2+^, but on perfusing the bath with 0.04 mm Ca^2+^, the fluid jet tip was filled with the low Ca^2+^ by sucking the bath solution back through its tip for 30 s (Corns et al., 2016). The effectiveness of the solution exchange was confirmed by additional experiments using bundle deflections with a glass probe. Furthermore, the increase in current in low Ca^2+^ in OHCs of *Tmc1*+/+ using the fluid jet (1.54 ± 0.10) agreed quantitatively with our previous measurements (1.52 ± 0.12) using only a glass probe stimulator (Beurg et al., 2006). For determining channel block by DHS, a similar approach to that with low Ca^2+^ was followed; results derived from fluid jet stimulation were confirmed with stimulation with a stiff glass probe and the points from two methods plotted as different symbols on the Hill plot. Patch electrodes were filled with the following: 142 mm CsCl, 3.5 mm MgCl_2_, 5 mm Na_2_ATP, 0.5 mm Na_2_GTP, 10 mm Tris phosphocreatine, 1 mm 1,2-bis(o-aminophenoxy) ethane-*N*,*N*,*N′*,*N′*-tetraacetic acid (BAPTA), and 10 mm Cs-HEPES, pH 7.2, and were connected to an Axopatch 200B amplifier. Electrode series resistances with 60% compensation were at best 3 MΩ, which together with a ∼5 pF cell capacitance gave recording time constants of 15 µs. Whole-cell currents were low-pass filtered with an eight-pole filter set to 5 kHz.

Stereociliary bundles were stimulated with a fluid jet from a pipette with a ∼10 µm in diameter tip or with a glass probe driven by a piezoactuator. The amplitude and time course of bundle motion was in some experiments calibrated by projecting the bundle image onto a pair of photodiodes and measuring the onset of the photocurrent (Crawford and Fettiplace, 1985; Ricci et al., 2005). Single MET-channel events were recorded in whole-cell mode after brief exposure of the bundle to saline containing 5 mm BAPTA plus 2.5 mm Ca^2+^ (Beurg et al., 2006, 2018). For all mutants, a subconductance state about half the amplitude of the full state was sometimes seen in a fraction of the traces (Beurg et al., 2018), but these events were not included in the amplitude histograms. Histograms of channel amplitude were fit with two Gaussians using a routine in IGOR Pro v8 (Wavemetrics). The Ca^2+^ selectivity of the MET channel relative to Cs^+^ was determined from Ca^2+^-reversal potentials of the MET current measured in an extracellular solution containing the following: 100 mm CaCl_2_, 20 mm N-methylglucamine, and 5 mm Tris, pH 7.4, and an intracellular Cs-based solution (see above). Reversal potentials were corrected for a −9 mV junction potential and were analyzed using the Goldman–Hodgkin–Katz (GHK) equation with activity corrections applied to the ion concentrations (Beurg et al., 2006; Kim et al., 2013). Experiments were performed at room temperature, 21–23°C.

#### Statistical test

All results are quoted as mean ± 1 SD and statistical tests used a two-tailed *t* test, significance tests indicated as: *p < 0.05, **p < 0.01, ***p < 0.001. ABRs and DPOAEs and immunologic labeling were performed on at least four mice for each mutation. MET currents were recorded and single channel conductance values were determined on four or more mice for each mutant. The FM1-43 assays for mechanotransduction were performed on two or three mice for each condition.

#### Next generation sequencing (NGS) of the proband on the OtoGenome panel

Targeted NGS was performed at the Laboratory for Molecular Medicine (Harvard Partners) on the OtoGenome platform (at the time of testing, the OtoGenome consisted of a targeted panel of 87 validated hearing loss genes) as previously described (Mandelker et al., 2014). DNA was extracted from peripheral blood lymphocytes using the Puregene genomic DNA isolation kit (QIAGEN). The gene panel test was performed by NGS using oligonucleotide-based target capture (Agilent SureSelect; Agilent Technologies, Santa Clara, CA), followed by Illumina HiSeq (Illumina Inc.) sequencing of the coding regions and splice sites of 87 genes associated with nonsyndromic hearing loss. Alignment and variant calling for single nucleotide variants and indels were determined with the Burrows–Wheeler Aligner and Genome Analysis Toolkit Unified Genotyper and copy number analysis was performed using an in-house developed tool (Pugh et al., 2016). Variants detected were assessed for pathogenicity and classified based on American College of Medical Genetics and Genomics criteria (Richards et al., 2015). Sanger sequencing was performed on family members as described previously (Zimmerman et al., 2010), using primers targeting exons 16–17 of *HsTMC1* (NM_138691.2) which encompass the c.1265C>A (p.T422K). The sequencing primers are: forward, 5′-CCAAAATTCTGGCAAAAAGC-3′ and reverse, 5′-CATGAAATTCAGAGCCAGCA-3′.

#### Localization of the deafness-causing mutations in TMC1

The TMC1 model was based on the *Nectria hematococca* TMEM16 structure (PDB ID: 4WIS; Ballesteros et al., 2018), but the intracellular TM6–TM7 loop containing residue W554 was not included in the original model because of the limited sequence homology with the template. We therefore modeled this intracellular loop in mTMC1 by using the loop modeling tools of I-TASSER (Yang et al., 2015) and SWISS-MODEL (Waterhouse et al., 2018), which build the loop by ab initio modeling. The global root mean square deviation (RMSD) between the two models was 3.5 Å for the 150 residues comprising the TM4–TM7 region as calculated by SuperPose (http://wishart.biology.ualberta.ca/SuperPose/), PROCHECK (Laskowski et al., 1993) was used to analyze the stereochemical quality of each model. The TMC1 model containing the TM6–TM7 loop generated by SWISS-MODEL presented better Ramachandran Plot statistics and lower G-factors to that generated by I-TASSSER, and was thus selected as the best model to depict residue W554. We used the PPM server (Lomize et al., 2012) to position and orient the TMC1 model in a lipid bilayer of adjustable thickness by minimizing its transfer energy from water to the membrane. The membrane building tool of CHARMM-GUI (Jo et al., 2008; Wu et al., 2014) was then used to generate and assemble the lipid bilayer and measure the pore size. UCSF Chimera v1.14 (Pettersen et al., 2004) software was used to visualize the structures and generate the final figures.

## Results

### Human *TMC1* p.T422K pathogenic variant

**Figure 1.**
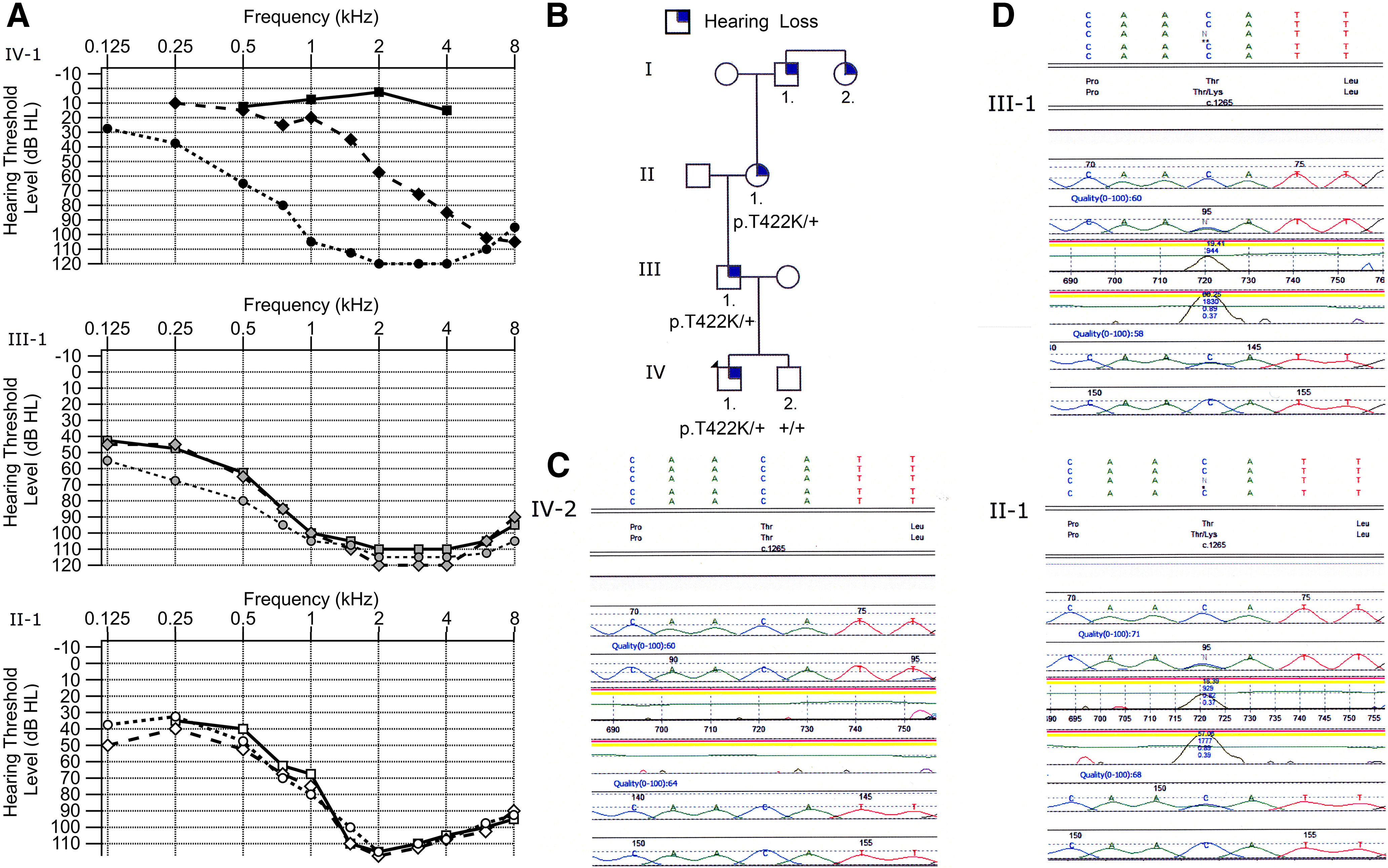
Genotype and phenotype of family. ***A***, Audiogram of the proband (top graph with filled in black markers), father (middle graph with filled in gray markers), grandmother (bottom graph with unfilled markers). Three time points are indicated (T1, rectangle; T2, diamond; T3, circle) for each individual. Each point on the graph is the average hearing threshold from the right and left ear. The proband was five years old at T1 and shows normal hearing at 0.5, 1, 2, and 4 kHz. At the second evaluation (T2), the patient was 11 years old and shows normal hearing from 0.25 to 1 kHz with hearing loss starting at 1.5 kHz. At T3, the patient was 14 years old and illustrates profound to severe hearing loss at all of the frequencies. The father shows moderate to profound hearing loss at all time points. Father was 37 years old at T1, 38 years old at T2, and 39 years old at T3. The grandmother also shows moderate to profound hearing loss at all frequencies. Her age was 57 years old at T1 and 58 years old at T2 and T3. ***B***, Pedigree of the family indicating autosomal dominant transmission pattern. Circles are indicative of a female and squares of a male. An individual with a filled in blue corner indicates that they report some degree of hearing loss. ***C***, DNA sequencing data for unaffected brother. ***D***, DNA sequencing data for father III-1 and grandmother II-1 both with hearing loss.

A new TMC1 missense pathogenic variant, *TMC1* p.T422K, was found in a family whose four-generation family history of hearing was evaluated (Fig. 1). The proband, an 11-year-old boy, presented with bilateral high frequency sensorineural hearing loss. He was born at term without complications and passed newborn hearing screening. Audiometric testing, at the time of presentation, demonstrated normal hearing from 250 to 1000 Hz, sloping to profound loss at 6000 Hz [Fig. 1*A*, IV-1, pure tone average (PTA) of both ears, dashed line]. Review of previous records showed a normal audiogram at five years of age at all four frequencies tested (0.5, 1, 2, and 4 kHz; Fig. 1*A*, solid line). At age 14, his audiogram showed moderate-severe hearing loss between 125 and 250 Hz and profound loss from 500 to 8000 Hz (Fig. 1*A*, IV-1 dotted line). He underwent unilateral left sided cochlear implantation, as hearing aids no longer provided benefit. He was otherwise healthy and met his developmental milestones on time and had no balance issues. However, his speech was notable for disarticulations suggesting long standing hearing loss. Genetic testing was performed using the OtoGenome panel, a targeted hearing loss panel that at the time, sequenced 87 known hearing loss genes. Testing identified three single heterozygous variants of unknown significance, specifically *OTOGL* c.2138C>A (p.A713D), *TMC1* c.1265C>A (p.T422k), and *TMIE* c.101C>T (p.T34M). As both OTOGL and TMIE are known as recessive genes, the likelihood of contributing to the proband's hearing loss was less as a second allele was not identified. *TMC1* mutations can be inherited as autosomal dominant as well as recessive. Threonine, an amino acid with a polar, uncharged side chain, is conserved in mouse, chicken, and platypus at this orthologous position (ensembl.org). In addition, the variant in *TMC1* was absent from large population databases (https://gnomad.broadinstitute.org/).

### Father of proband (III-1)

The proband's father was 37 years old at the time of presentation. He was diagnosed with hearing loss at age 15 after failing a school hearing screening examination. Review of audiograms from three time points over a three-year period from age 37 to 39 were essentially unchanged and showed down sloping moderate-severe to profound hearing loss (Fig. 1*A*, III-1). He underwent cochlear implantation at age 39. Allele-specific genetic testing showed that he had the same heterozygous variant in *TMC1,* c.1265C>A (p.T422K; Fig. 1*D*, III-1).

### Grandmother of proband (II-1)

The proband's paternal grandmother was 56 years old at the time of presentation. She reported bilateral hearing loss starting in her early twenties with progression over time. Audiograms at three time points, between ages 57 and 58, revealed PTA in the moderate to profound range from 125 to 1500 Hz with no responses from 2000–8000 Hz (Fig. 1*A*, II-1). She underwent cochlear implantation at age 58. Allele-specific genetic testing showed that she had the same heterozygous variant in *TMC1,* c.1265C>A (p.T422K; Fig. 1*D*, II-1).

### Brother of proband (IV-2)

The proband's brother was 12 years old at the time of evaluation and no hearing abnormalities were reported. He passed the newborn hearing screening, and had normal audiograms at 3, 9, and 10 years. Allele-specific genetic testing showed bi-allelic normal copies of *TMC1* (Fig. 1*C*).

### Murine *Tmc1* p.T416K deafness-causing mutation

**Figure 2.**
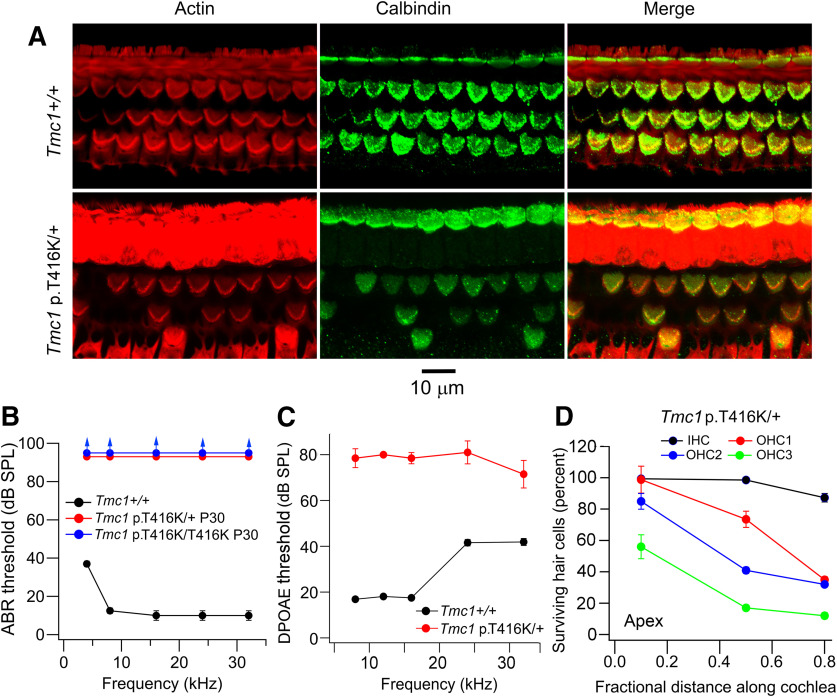
Auditory phenotype of mouse *Tmc1* p.T416K; *Tmc*2+/+. ***A***, Fixed whole mounts of mid-region of cochleas of P30 *Tmc1*+/+ (top row) and *Tmc1* p.T416K/+ (bottom row) labeled with phalloidin (Actin), calbindin28K (cell somas) and merge. Note OHC loss in mutant. ***B***, ABRs thresholds of P30 wild-type, *Tmc1* p.T416K/T416K homozygote, and *Tmc1* p.T416K/+ heterozygote showing dominant phenotype. ***C***, DPOAEs thresholds for *Tmc1*+/+ (black symbols, *N* = 6) and *Tmc1* p.T416K/+ (red symbols, *N* = 8). ***D***, Hair cell survival in P30 *Tmc1* p.T416K/+ heterozygote. For each cell type, ∼100 cells along the cochlea were included in the count to construct average. In each panel, the mean +/− SD is plotted.

A mouse model carrying the homologous mutation of *TMC1* p.T422K, *Tmc1* p.T416K, was generated using CRISPR/Cas9 technology (see Materials and Methods) and characterized using hair cell survival (Fig. 2*A*), ABRs (Fig. 2*B*), and otoacoustic emissions (Fig. 2*C*). ABRs revealed profound hearing loss across the entire frequency spectrum in both homozygotes and heterozygotes at P30, consistent with the dominance of the human mutation. Degeneration and absence of OHCs was evident at P30 (Fig. 2*A*,*D*), with the base being more affected than the apex at that age, as with many cochlear insults (Fettiplace and Nam, 2019). Despite loss of fewer OHCs at the apex, it is likely that the cells remaining were nonfunctional since the threshold of DPOAEs, which are a measure of OHC functionality as nonlinear amplifiers (Kemp, 2002), were elevated throughout the frequency range and by 60 dB at low frequencies (Fig. 2*C*). There was loss of fewer IHCs compared with OHCs (Fig. 2*A*,*D*), although as described later, all hair cells were probably non-transducing at P30.

**Figure 3.**
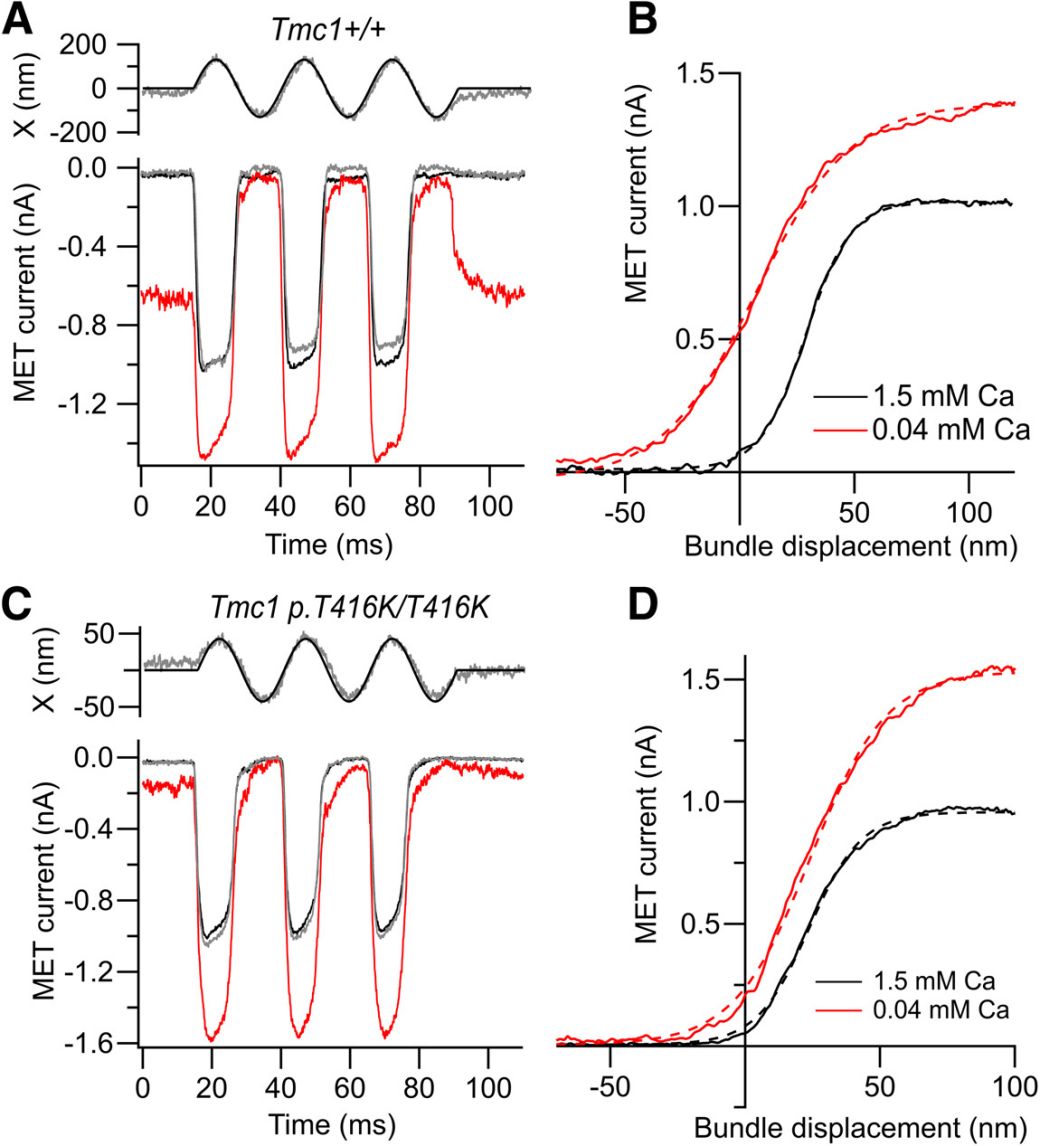
MET currents of apical OHCs for control and p.T416K mutant mice. ***A***, MET currents in response to bundle vibration in *Tmc1*+/+*; Tmc2*−/− in saline containing 1.5 mm Ca^2+^ (black, gray before and after) and endolymph-like 0.04 mm Ca^2+^ (red); top trace is driving voltage to piezo (smooth curve) superimposed on photodiode signal (noisy gray trace). ***B***, Current-displacement relations evaluated from responses in ***A*** for 1.5 mm Ca^2+^ (black) and 0.04 mm Ca^2+^ (red). Lowering external Ca^2+^ increased maximum current I_MAX_ and fraction on at rest. Dashed curves are fits of current I against displacement x to Boltzmann equation: I = I_MAX_/[1 + exp((x1 – x)/a)], where I_MAX_ = 1.0 nA, a = 9.8 nm, x1 = 29 nm (1.5 Ca^2+^) and I_MAX_ = 1.43 nA, a = 21 nm, x1 = 6.9 nm (0.04 Ca^2+^). ***C***, MET currents in response to bundle vibration in *Tmc1* p.T416K/T416K*; Tmc2*−/− mice in saline containing 1.5 mm Ca^2+^ (black, gray before and after) and endolymph-like 0.04 mm Ca^2+^ (red); top trace is driving voltage to piezo (smooth curve) superimposed on photodiode signal (noisy gray trace). ***D***, Current-displacement relations evaluated from responses in ***C*** for 1.5 mm Ca^2+^ (black) and 0.04 mm Ca^2+^ (red). Dashed curves are fits to Boltzmann equation, where I_MAX_ = 0.98 nA, a = 10.4 nm, x1 = 23 nm (1.5 Ca^2+^) and I_MAX_ = 1.56 nA, a = 14.7 nm, x1 = 23.6 nm (0.04 Ca^2+^). Note that in mutant, low Ca^2+^ still increases maximum current but has smaller effect on resting open probability, and no effect on x1, i.e., no shift in the activation curve; holding potential −84 mV. The difference in resting open probability between control, and mutant was not seen in 1.5 mm extracellular Ca^2+^ (see text).

The consequences of the p.T416K mutation for transduction were studied by recording MET currents at the end of the first postnatal week when MET channel development was complete and currents were maximal, but the hair bundles still appeared normal (Beurg et al., 2018). Measurements were taken on a *Tmc2*−/− background to extract the specific transducer effects of the mutation and rule out the contribution of TMC2. In extracellular 1.5 mm Ca^2+^ the maximum MET current in apical OHCs of *Tmc1* p.T416K/T416K homozygotes was 1.15 ± 0. 10 nA (*N* = 18), not significantly different from the control current in *Tmc1*+/+ mice, which was 1.18 ± 0.16 nA (*N* = 14; Fig. 3). The maximum MET current in basal OHCs of the mutant was 1.39 ± 0.08 nA (*N* = 5), significantly larger than the current in apical OHCs (*t* test, *p* < 0.0005), suggesting that the tonotopic gradient was still present in the mutant. When extracellular Ca^2+^ was reduced to 0.04 mm, as in endolymph (Bosher and Warren, 1978; Ikeda et al., 1987), the maximum current increased by 50% in both genotypes and a large fraction of the current was turned on at the resting position of the bundle. In the control, the fraction of current open at rest, P_OR_, increased as previously reported (Johnson et al., 2011) to 0.44 ± 0.06 (*N* = 8), but in the *Tmc1* p.T416K/T416K homozygote, the increase in P_OR_ was smaller, to 0.22 ± 0.06 (*N* = 6), significantly different from the control (*t* test, *p* < 0.001; Fig. 3). The difference in resting open probability was not observed in the 1.5 mm extracellular Ca^2+^: in *Tmc1*+/+, P_OR_ = 0.030 ± 0.01 (*N* = 8), whereas in *Tmc1* p.T416K/T416K, P_OR_ = 0.037 ± 0.03 (*N* = 6; difference not significant: *t* test, *p* = 0.45). A smaller increase in P_OR_ in 0.04 mm was also seen in *Tmc1* p.M412K/M412K, where the P_OR_ was 0.19 ± 0.07 (*N* = 7; Beurg et al., 2015a), suggesting that open channels are less stable at rest.

**Figure 4.**
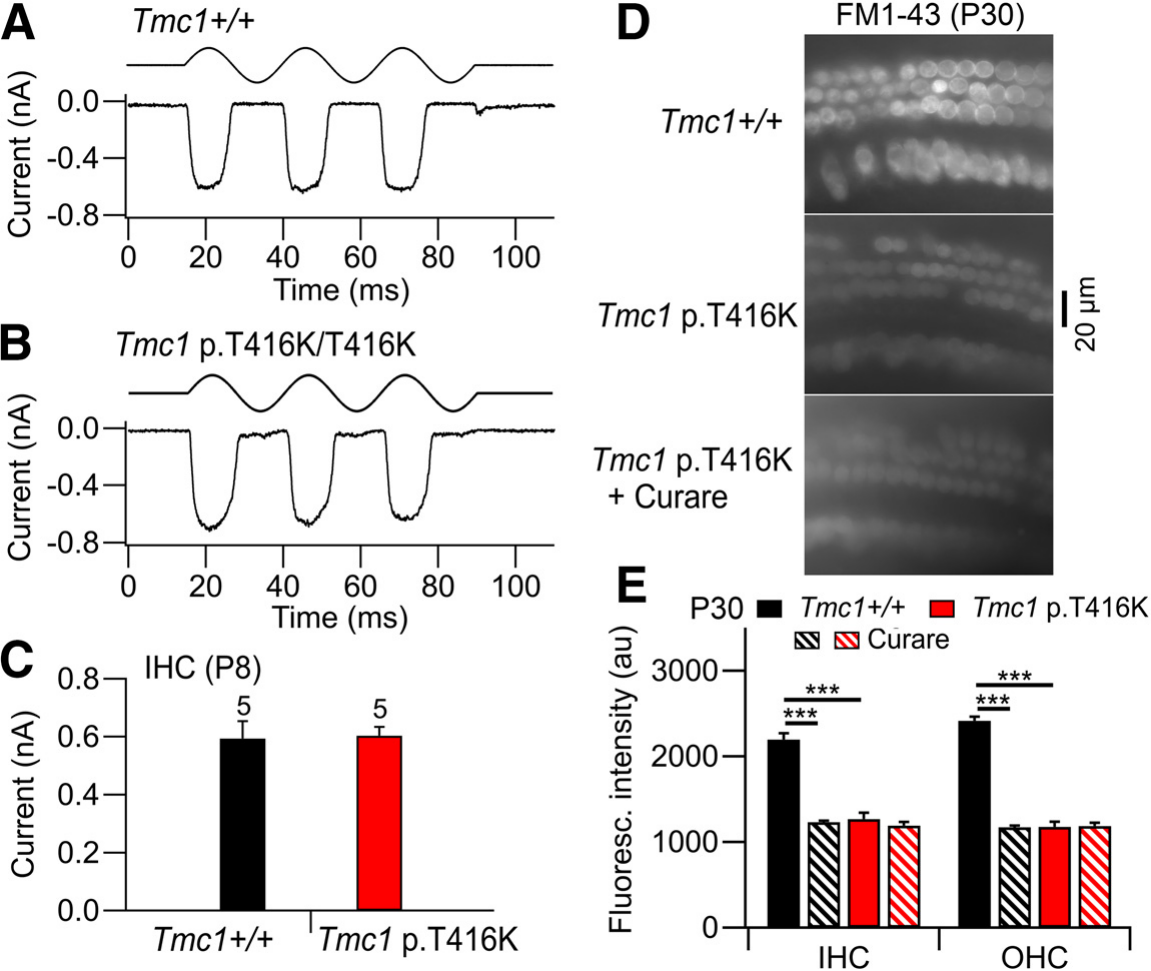
MET currents in IHCs of control and p.T416K mutant mice. ***A***, MET current for *Tmc1*+/+*; Tmc2*−/− in response to sinusoidal motion of hair bundle. ***B***, MET current for *Tmc1* p.T416K/T416K*; Tmc2*−/−. ***C***, Collected results of MET current amplitudes in five control (black) and five p.T416K/T416K mutant (red) P8 IHCs. For ***A***–***C***, holding potential −84 mV. ***D***, Hair cell fluorescence in unfixed cochleas of P30 mice indicating FM1-43 entry in apical hair cells of *Tmc1*+/+*; Tmc2*−/− (top), and *Tmc1* p.T416K/T416K*; Tmc2*−/− mice in the absence (middle) and presence (bottom) of 0.1 mm *d*-tubocurarine (curare) to block MET channels. In the mutant, OHCs are shrunken and some are missing. ***E***, Collected results showing the fluorescence intensity (au) in 36 IHCs and 40 OHCs in two preparations. *Tmc1*+/+ and *Tmc1* p.T416K were significantly different (***p < 0.001) for IHCs and OHCs but *Tmc1* p.T416K with and without *d*-tubocurarine were not significantly different (*p* = 0.28 IHCs, *p* = 0.18 OHCs). Results indicate functional channels that can be blocked by curare in *Tmc1*+/+ but not *Tmc1* p.T416K. In panels ***C*** and ***E***, bars denote mean +/− SD.

MET currents were also of normal amplitude in IHCs of *Tmc1* p.T416K at P8 (Fig. 4*A*,*B*) and not significantly different from *Tmc1*+/+ control (Fig. 4*C*). The transducing ability of both IHCs and OHCs was assessed at P30 using influx of FM1-43 as a marker of intact MET channels (Gale et al., 2001). The assay was performed on apical hair cells in an unfixed cochlear explant (see Materials and Methods). The fluorescent signal in *Tmc1*+/+ hair cells because of FM1-43 entry was approximately halved if the MET channels were blocked with 0.1 mm *d*-tubocurarine (Glowatzki et al., 1997; Krey et al., 2020). Cell fluorescence was also reduced in both IHCs and OHCs of *Tmc1* p.T416K relative to *Tmc1*+/+ (Fig. 4*D*,*E*). However, the residual fluorescence in *Tmc1* p.T416K persisted in the presence of *d*-tubocurarine (Fig. 4*E*), indicating that MET channels in both types of hair cell are absent in the mutant at P30, corroborating the ABR measurements.

**Figure 5.**
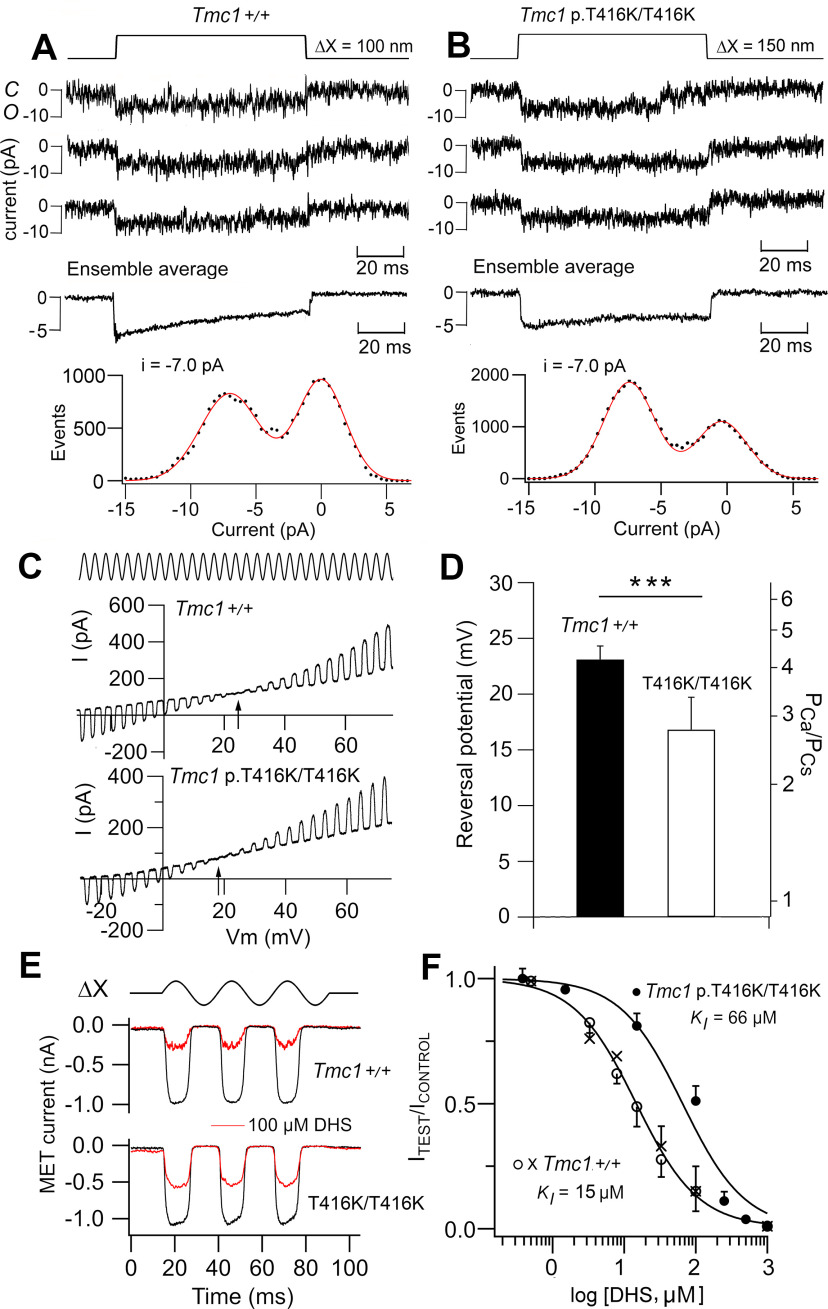
Ionic properties of MET channels from apical OHCs of *Tmc1* p.T416K/T416K*; Tmc2*−/−. ***A***, Three examples of single MET channel currents for *Tmc1*+/+*; Tmc2*−/−, ensemble average of 30 presentations (middle) and amplitude histogram (bottom) giving −7.0 pA at –84 mV. C and O indicate closed and open states of channel. ***B***, Examples of single MET channel currents for *Tmc1* p.T416K/T416K*; Tmc2*−/−, ensemble average of 100 presentations (middle) and amplitude histogram (bottom) giving −7.0 pA at −84 mV. ***C***, Determination of MET channel Ca^2+^ permeability in isotonic external Ca^2+^ and internal Cs^+^ (see Materials and Methods). During a sinusoidal bundle vibration (ΔX, ±200 nm), the membrane potential (V_m_) was swept from −30 to +80 mV and the MET current reversed polarity at arrowed potential. Reversal potential in *Tmc1*+/+*; Tmc2*−/− is ∼7 mV positive to that in *Tmc1* p.T416K/T416K*; Tmc2*−/−. ***D***, Collected reversal potentials and relative permeability P_Ca_/P_Cs_ in six cells mean +/− SD. Control and mutant are significantly different (*t* test, ***p < 0.001). ***E***, MET currents for bundle vibrations (ΔX, ±200 nm) in normal saline (black traces) and in presence of 100 μm DHS in *Tmc1*+/+*; Tmc2*−/− (top) and *Tmc1* p.T416K/T416K*; Tmc2*−/− (bottom). ***F***, Mean ± SD (*N* = 5) of MET current block by DHS in *Tmc1*+/+ and *Tmc1* p.T416K/T416K. Hill plots give K_I_ = 15 μm (all points) and 66.0 μm, respectively, with Hill coefficients = 1 for both genotypes. For control curve, open symbols determined using bundle stimulation with a fluid et (K_I_ = 14.0 μm), whereas crosses obtained with a stiff glass probe (K_I_ = 15.4 μm). The similar K_I_ values for the different jstimulation methods rules out artifacts because of dilution of DHS by saline in the fluid jet.

The elementary channel conductance was determined from recording of single channels after severing most of the tip links with submicromolar Ca^2+^ (Beurg et al., 2006, 2015b). All measurements were performed on *Tmc2*−/− mice. Recordings from apical OHCs (Fig. 5*A*,*B*) gave a unitary current in the OHCs illustrated of −7.0 pA in *Tmc1*+/+ control and *Tmc1* p.T416K/T416K mutant. The mean single-channel conductance averaged over all cells was 84.8 ± 3.1 pS (*N* = 5) for control and 87.2 ± 4.5 pS (*N* = 4) for the mutant (differences not significant; *t* test, *p* = 0.35). Thus, the mutation had no effect on the single-channel conductance. The Ca^2+^ permeability of the MET channel and its susceptibility to block by DHS were also determined and both were reduced in apical OHCs of the *Tmc1* p.T416K/T416K. The Ca^2+^ permeability relative to Cs^+^ (P_Ca_/P_Cs_) of the MET channel was determined from the reversal potential of the current in isotonic Ca^2+^ saline and the permeability was inferred with the GHK equation (Beurg et al., 2006; Kim et al., 2013). The Ca^2+^ permeability relative to Cs^+^ in *Tmc1* p.T416K/T416K was 2.80 ± 0.5 (*N* = 6) compared with 4.25 ± 0.35 (*N* = 8) in the control *Tmc1*+/+, the difference being significant (*t* test, *p* = 0.0004; Fig. 5*C*,*D*). Another property of the *Tmc1* p.T416K/T416K channels was diminished efficacy of channel block by the aminoglycoside antibiotic DHS, known to enter and block the wild-type MET channel at micromolar concentrations (Marcotti et al., 2005). The half-blocking concentration for DHS was 66 ± 13 μm (four cells per dose) in the *Tmc1* p.T416K/T416K mutant mice and 15 ± 0.7 μm (four cells per dose) for the *Tmc1*+/+ control mice (Fig. 5*E*,*F*). Effects on both parameters, a reduction in Ca^2+^ permeability and decreased potency of extracellular DHS, were also observed in the mutation *Tmc1* p.M412K/M412K (Beurg et al., 2015a; Corns et al., 2016).

### *Tmc1* p.D528N and *Tmc1* p.W554L mutations

**Figure 6.**
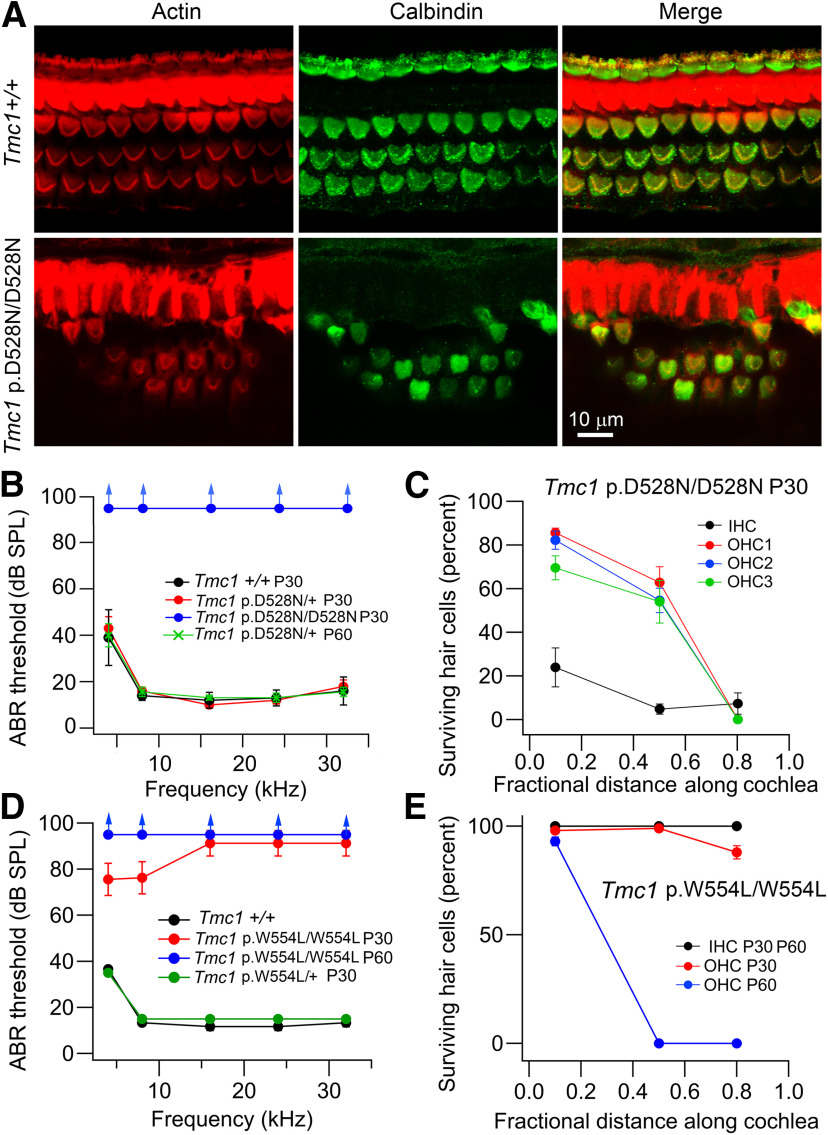
Deafness phenotypes in *Tmc1* p.D528N and *Tmc1* p.W554L. ***A***, Fixed whole mounts of mid-region of cochleae of P30 *Tmc1*+/+ (top row) and *Tmc1* p.D528N/D528N (bottom row) labeled with phalloidin (actin), calbindin28K (cell somas) and merge. Note absence of IHCs and loss of OHCs, and shape changes to OHC hair bundle in mutant. ***B***, Mean ABR thresholds for four P30 mice in wild-type *Tmc1*+/+, *Tmc1* p.D528N/+, and *Tmc1* p.D528N/D528N; *Tmc2*+/+ mice. The similarity of wild type and heterozygote indicates recessive mutation. ***C***, Survival of IHC and OHC (rows 1, 2, and 3) in four P30 mice in *Tmc1* p.D528N/D528N; *Tmc2*+/+ mice. ***D***, ABR thresholds for four P30 mice in wild-type *Tmc1*+/+, *Tmc1* p.W554L*/+* at P30 and *Tmc1* p.W554L/W554L at P30 and P60 (all on *Tmc2*+/+ background). This mutation is recessive too. ***E***, Survival of IHC and OHCs in four P30 mice in *Tmc1* p.W554L/W554L; *Tmc2*+/+ mice at P30 and P60. At P60, only OHCs are missing.

We characterized the transducing abilities in two other *Tmc1* mouse mutants, *Tmc1* p.D528N and *Tmc1* p.W554L. *Tmc1* p.D528N is a new missense mutation leading to the substitution of an asparagine for the negatively charged aspartate in TM6 using the current model of TMC1 (Ballesteros et al., 2018). *Tmc1* p.W554L mutation was previously reported as the *stitch* mutant mouse arising from N-ethyl-N-nitrosourea mutagenesis (Manji et al., 2012), and affects an aromatic residue located at the intracellular loop between TM6 and TM7. Both were recessive deafness-causing mutations (Fig. 6) but had different severity. In *Tmc1* p.D528N mutant mice, there was no ABR at P30 and there was loss of cochlear hair cells, more prominent in basal OHCs and throughout the cochlea in IHCs (Fig. 6*A*,*B*). In the *Tmc1* p.W554L mutants the ABR had some low frequency response at P30, but no response was evident at P60 (Fig. 6*D*). There was also no IHC or OHC loss at P30 but basal and mid-region OHCs had degenerated by P60 (Fig. 6*E*). The delayed effects on the ABR and OHC loss both indicate that the *Tmc1* p.W554L mutation is progressive.

**Figure 7.**
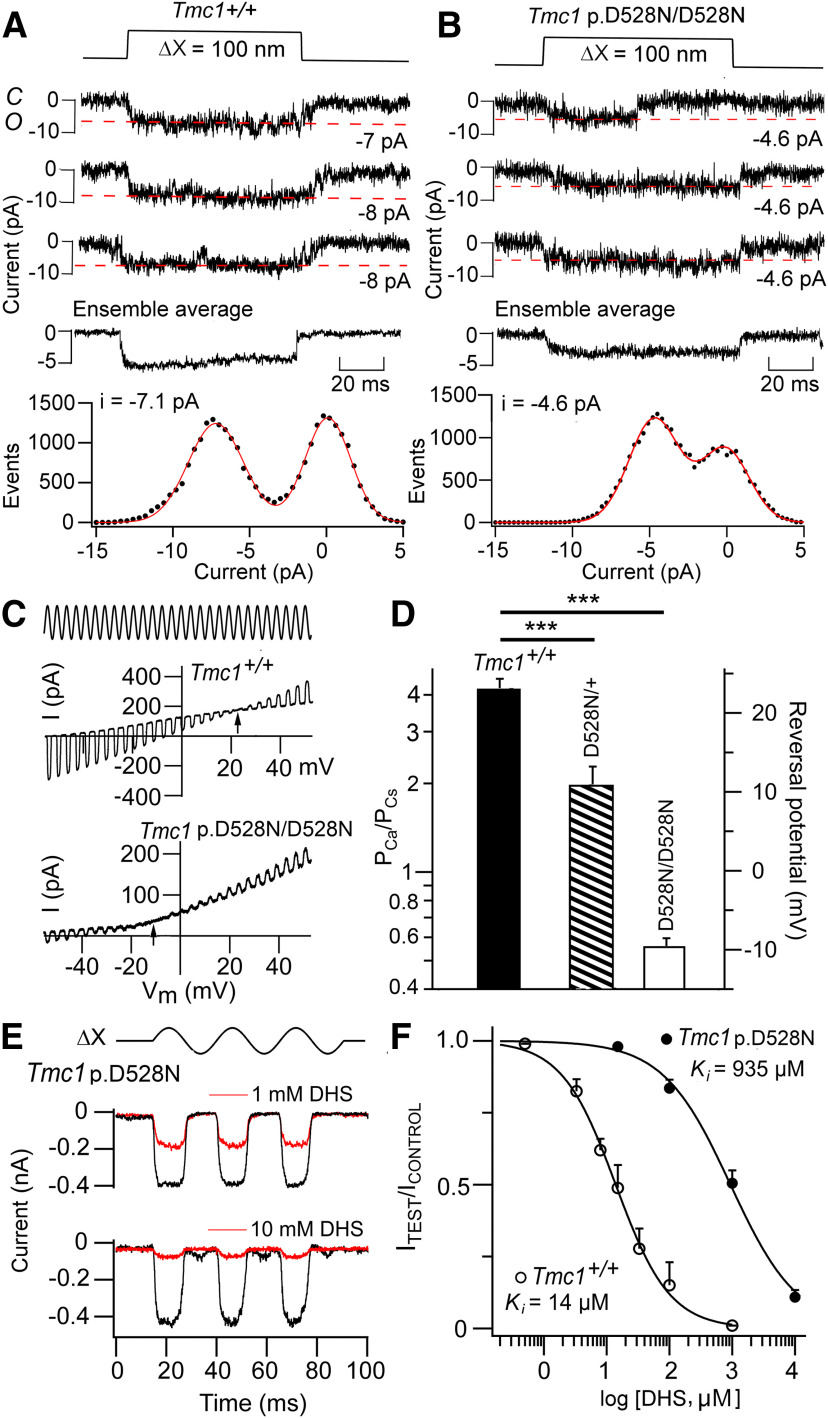
Ionic properties of MET channels from apical OHCs of *Tmc1* p.D528N/D528N*; Tmc2*−/−. ***A***, Three examples of single MET channel currents for *Tmc1*+/+*; Tmc2*−/−, ensemble average of 50 presentations (middle) and amplitude histogram (bottom) giving −7.1 pA at −84 mV. ***B***, Examples of single MET channel currents for *Tmc1* p.D528N/D528N*; Tmc2*−/−, ensemble average of 30 presentations (middle) and amplitude histogram (bottom) giving −4.6 pA at −84 mV, significantly smaller than control (*p* < 0.001). ***C***, Determination of MET channel Ca^2+^ permeability in isotonic external Ca^2+^ and internal Cs^+^ (see Materials and Methods). During a sinusoidal bundle vibration (ΔX, ±200 nm), the membrane potential (V_m_) was swept from −80 to +80 mV and the MET current reversed polarity at arrowed potential. Reversal potential in *Tmc1* p.D528N/D528N*; Tmc2*−/− is ∼34 mV negative to *Tmc1*+/+*; Tmc2*−/− indicating much reduced Ca^2+^ permeability. ***D***, Collected reversal potentials and relative permeability P_Ca_/P_Cs_ in four cells each of *Tmc1*+/+, *Tmc1* p.D528N*/+* and *Tmc1* p.D528N/D528N. All of which are significantly different (***) *p* < 0.001. Note the heterozygote reversal potential lies between wild type and homozygote. ***E***, MET currents for bundle vibrations (ΔX, ±200 nm) in normal saline (black traces) and on adding DHS at 1 mm (top) and 10 mm (bottom) in *Tmc1* p.D528N/D528N*; Tmc2*−/− (bottom). ***F***, Mean ± SD (*N* = 5) of MET current block by DHS in *Tmc1*+/+ (*N* = 5) and *Tmc1* p.D528N/D528N (*N* = 3). Hill plots give K_I_ = 14 μm, Hill coefficient = 1 for control and 935 μm, Hill coefficient = 0.81 for mutant.

Mechanotransduction was assayed in apical OHCs of *Tmc1* p.D528N mice at the end of the first postnatal week P5–P7. Several significant changes were found including a large reduction in Ca^2+^ permeability and importantly a reduction in single-channel conductance. Single channel currents were recorded in OHCs (Fig. 7*A*,*B*), those illustrated having amplitudes at −84 mV of 7.1 pA in the *Tmc1*+/+ control and 4.6 pA in the *Tmc1* p.D528N/D528N mutant. Mean conductance values were 84.8 ± 3.1 pS (*N* = 5) in the control and 53.5 ± 2.9 pS (*N* = 5) in the mutant, a significant difference (*t* test, *p* = 0.001) corresponding to a 37% reduction in conductance relative to the control. The macroscopic MET current was about half the size of the controls (0.55 ± 0.05 nA, *N* = 11; difference significant, *p* = 0.001) in apical OHCs and smaller (0.39 ± 0.11 pA, *N* = 3) in P8 *Tmc1* p.D528N/D528N control IHCs than in *Tmc1*+/+ controls IHCs (0.59 ± 0.06 pA, *N* = 5; Fig. 4*C*). The reduced macroscopic current is largely explained by smaller single-channel conductance rather than by a reduced number of channels in the bundle. The Ca^2+^ permeability (P_Ca_/P_Cs_; Fig. 7*C*,*D*) was reduced 7-fold relative to control from 4.25 ± 0.35 (*N* = 8) to 0.57 ± 0.04 (*N* = 7) in the homozygotes, *Tmc1* p.D528N/D528N. We also characterized the heterozygote *Tmc1* p.D528N*/+;* and found an intermediate reduction to 2.00 ± 0.33 (*N* = 6), which is about half-way between the wild type and homozygote. Despite the large decrease in Ca^2+^ permeability, there was no effect on the external Ca^2+^ block, and the current amplitude increased to 1.48 ± 0.19 (*N* = 4) on lowering external Ca^2+^ from 1.5 mm to 0.04 mm. Nor was there a large effect of the mutation on the P_OR_, which was 0.39 ± 0.01 (*N* = 4) in the homozygote.

Compared with *Tmc1* p.T416K/T416K, there was an even larger 67-fold reduction in the effectiveness of DHS to block the MET channel in *Tmc1* p.D528N/D528N. The half-blocking concentration for DHS was 925 ± 95 μm (*N* = 3 cells per dose) for the homozygous p.D528N mutant compared with 15 ± 0.7 μm (*N* = 4 cells per dose) for the control *Tmc1*+/+ mice (Fig. 7*E*,*F*). Both the reduction in Ca^2+^ permeability and decreased potency of extracellular DHS were seen in the *Tmc1* p.M412K/M412K mutation (Beurg et al., 2015a; Corns et al., 2016), but the effects of p.D528N were in both cases much larger. The effect of p.D528N may be partly attributable to restricting DHS access into the pore.

**Figure 8.**
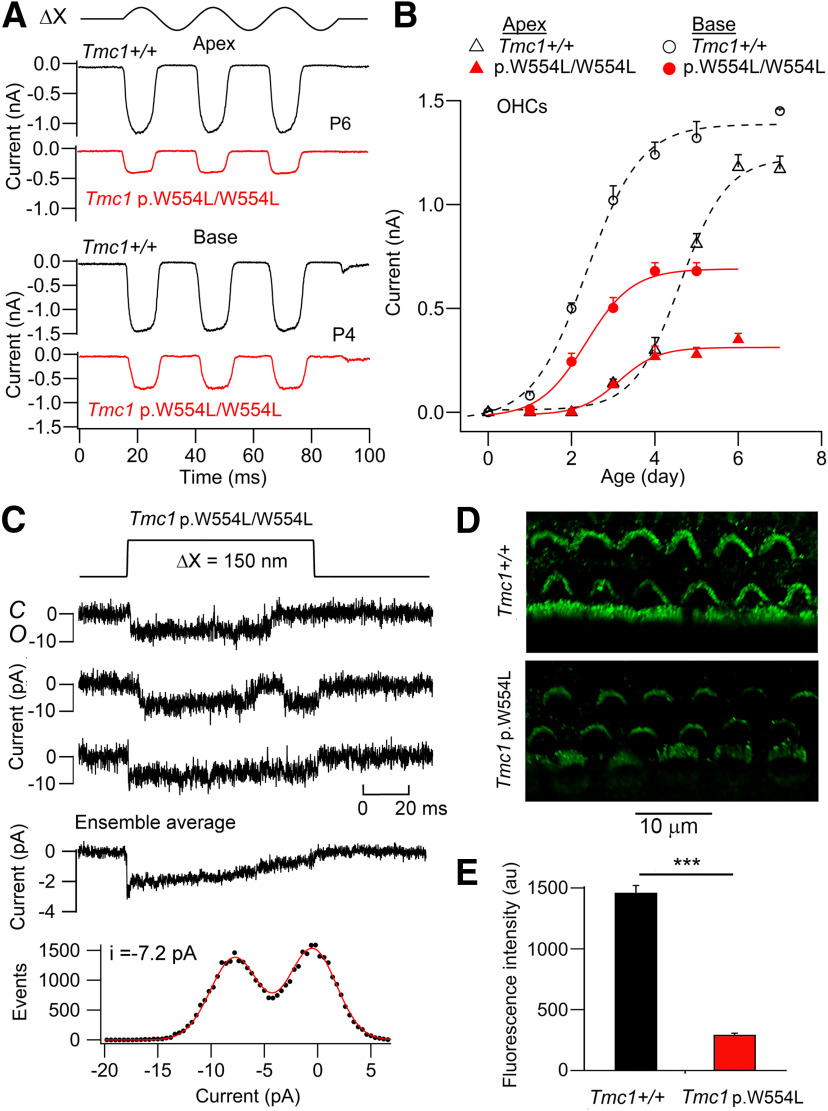
MET currents from OHCs of *Tmc1* p.W554L/W554L*; Tmc2*−/−. ***A***, Maximum currents in *Tmc1*+/+ (black traces) and *Tmc1* p.W554L/W554L (red traces) for hair bundle vibrations (ΔX, ±200 nm) in apical and basal OHCs. ***B***, Neonatal development of MET current (mean ± SD, *N* = 6) for basal and apical OHCs. Note the basal OHC currents develop about 2 d earlier and reach a larger maximum amplitude. The tonotopic gradient is preserved in the mutant but the maximum currents are up to 6-fold smaller. ***C***, Examples of single MET channel currents for *Tmc1* p.W554L/W554L*; Tmc2*−/−, ensemble average of 30 presentations (middle) and amplitude histogram (bottom) giving single channel −7.2 pA. Holding potential −84 mV. ***D***, TMC1 immunolabeling of apical hair cells in P6 mice of *Tmc1*+/+*; Tmc2*−/− (top) and *Tmc1* p.W554L/W554L*; Tmc2*−/− (bottom). ***E***, Collected measurements of fluorescent intensity (au) in *Tmc1*+/+*; Tmc2*−/− and *Tmc1* p.W554L/W554L*; Tmc2*−/−. Bars denote mean +/− SD. Mean values are significantly different *p* < 0.001 (***). Measurements made on 115 cells (control) and 75 cells (mutant) of four mice.

The consequences of the missense mutation *Tmc1* p.W554L/W554L were less severe than for the other mutants studied. There was a reduction in the maximum current, up to 60%, at both the apex and base (Fig. 8*A*,*B*). The maximum MET current in OHCs developed over the first week, becoming maximal at P4 at the base of the cochlea and P6 at the apex. Unlike *Tmc1* p.D569N (Beurg et al., 2019), tonotopy was preserved and the maximum current although reduced was still larger at the base than at the apex. In contrast, there was no effect on single channel current (Fig. 8*C*), the mean unitary conductance being 88.3 ± 4.0 pS (*N* = 4), not significantly different from *Tmc1*+/+ 84.8 ± 3.1 pS (*N* = 5; *t* test, *p* = 0.52). Furthermore, there was only a modest change in Ca^2+^ permeability with P_Ca_/P_Cs_ = 3.59 ± 0.27 (*N* = 8). The reduction in maximum MET current in the mutant is attributable to a decrease in the channel expression at the transduction site. The number of functional MET channels was inferred from the ratio of the macroscopic to single-channel currents. Thus, the number of active MET channels was 58 ± 13 (*N* = 4) in *Tmc1* p.W554L/W554L compared with 166 ± 23 (*N* = 5) in *Tmc1*+/+. The reduction in channel number was confirmed by immunolabeling for TMC1 (Fig. 8*D*), showing bundle labeling intensity was reduced 5-fold, a significant change (*t* test, *p* = 0.001) in *Tmc1* p.W554L/W554L compared with *Tmc1*+/+ (Fig. 8*E*).

### The timetable for transduction loss

**Figure 9.**
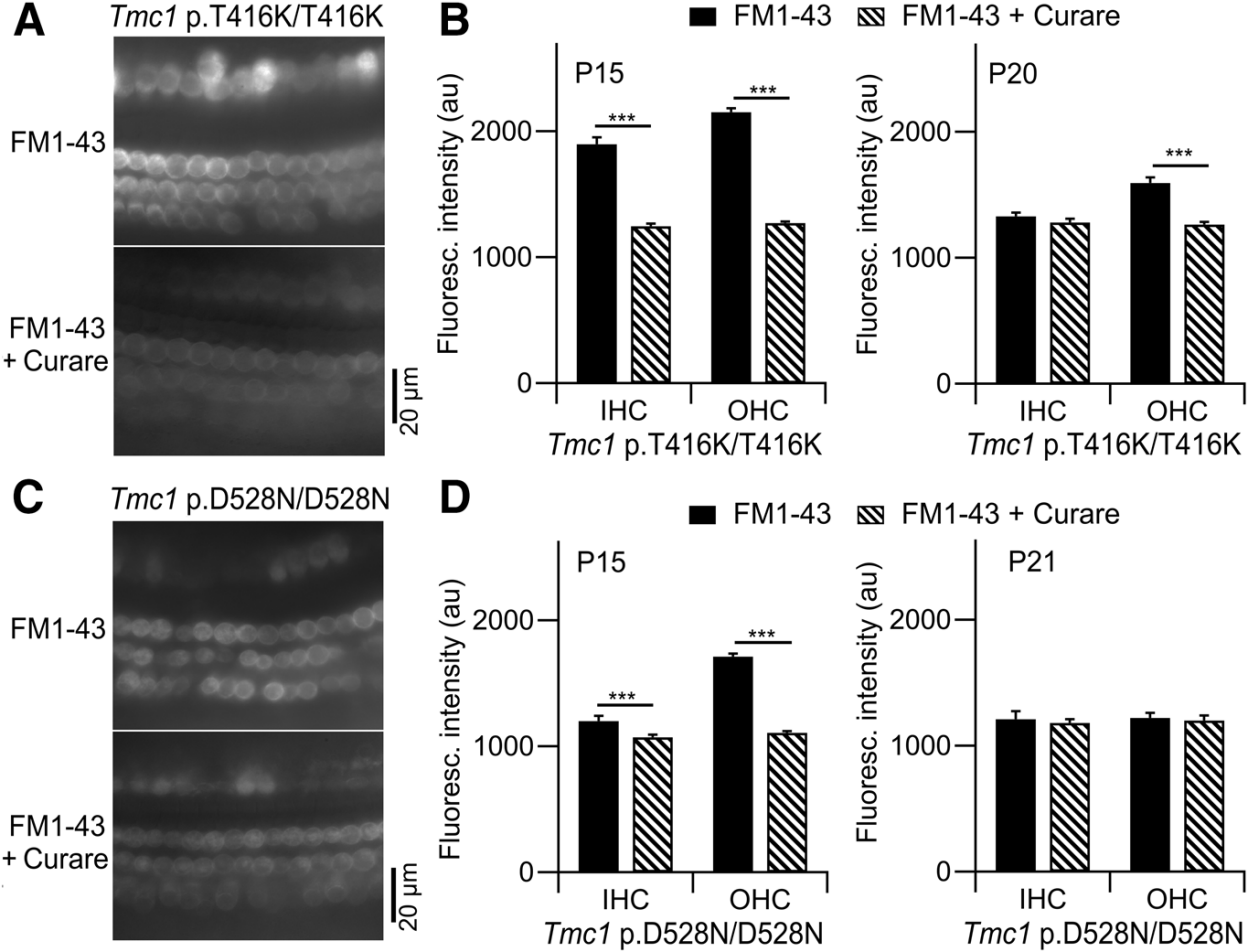
Loss of mechanotransduction determined with FM1-43 influx. ***A***, Fluorescence images showing one row of IHCs and three rows of OHCs in the apical turn of cochlear explants of P15 *Tmc1* p.T416K/T416k*; Tmc2*−/− mice after exposure to 6 μm FM1-43 in the absence (top) and presence (bottom) of 0.1 mm curare. ***B***, Fluorescence intensity in IHCs and OHCs with and without *d*-tubocurarine in P15 mice (left) and P21 mice (right). Number of cells measured (±curare): P15 IHCs 76, 65; P15 OHCs 120, 145; P21 IHCs 55, 50; OHCs 69, 72. ***C***, Fluorescence images in apical turns of P15 *Tmc1* p.D528N/D528N*; Tmc2*−/− mice after exposure to 6 μm FM1-43 in the absence (top) and presence (bottom) of 0.1 mm curare. ***D***, Fluorescence intensity in IHCs and OHCs ± curare in P15 mice (left) and P21 mice (right). Number of cells measured (±curare): P15 IHCs 81, 80; P15 OHCs 278, 178; P21 IHCs 25, 24; OHCs 68, 61. The small number of P21 IHCs reflect loss of these cells. All IHC measurements were made at slightly different focal plane to OHCs; *** statistical significance indicated *t* test, *p* < 0.001, in panles ***B–D***, bars denote mean +/− SD.

The time course for disappearance of mechanotransduction was documented in *Tmc1* p.T416K/T416K and in *Tmc1* p.D528N/D528N to address possible mechanisms underlying hair cell loss in the *Tmc1* mutants. Based on electrophysiological recordings, both genotypes are known to possess functional MET channels in the early postnatal period at P5–P8 (Figs. 3, 4, 7), but based on ABR measurements, those mice were deaf by P30 (Figs. 2*B*, 6*B*). We examined transduction in the third postnatal week, after the onset of hearing at P12. It was difficult to use ABRs on mice of this age range, so instead we employed the Preyer (pinna twitch) reflex to test whether hearing was present (see Materials and Methods). With this method, *Tmc1* p.T416K/T416K were all still hearing at P15 (6/6 mice positive reflex) but not at P20 (0/4 mice positive), whereas some *Tmc1* p.D528N/D528N mice were deaf at P15 (5/9 mice positive reflex) and all at P21 (0/8 mice positive). To determine whether deafness was associated with loss of mechanotransduction at these ages, hair cell fluorescence because of the influx of FM1-43 through the MET channels was used to reveal functional channels (Gale et al., 2001; Meyers et al., 2003; Fig. 9). In these experiments, FM1-43 was perfused into the apical turn of temporal bone explants in the presence or absence of MET channel blockers; the solution perfused was ice cold to minimize energy-dependent uptake of the dye by endocytic processes (Vélez-Ortega et al., 2017). For *Tmc1* p.T416K/T416K mice, FM1-43 fluorescence was partially suppressed in IHCs and OHCs in P15 cochleas after blocking the channels with 0.1 mm *d*-tubocurarine; this finding shows that both hair cell types transduce at P15. However, by P21, significant transduction was present in OHCs but not in IHC (Fig. 9*B*). For *Tmc1* p.D528N/D528N mice, significant transduction occurred in both IHCs and OHCs at P15, but in neither cell type by P21 (Fig. 9*D*). We conclude from these observations that mechanotransduction was lost in the mutants between P15 and P21. At this time in wild-type mice, the endocochlear potential attains its maximal value (Steel and Barkway, 1989) and hearing sensitivity grows toward its adult level (Ehret, 1976).

### Conclusions

The properties of functional MET channels in the five mutations, characterized at P6 in apical OHCs, are collected in Table 1 and include single-channel conductance, Ca^2+^ permeability and the numbers of “active” channels, N_MET_. The damage to, or loss of, cells at P30 is directly related to the decrease in Ca^2+^ permeability. In order of severity, D528N > D569N > M412K > T416K > W554L. N_MET_ represents the number of MET channels recruitable by bundle stimulation and was determined as the ratio of the macroscopic to single-channel conductance. It is noteworthy that N_MET_ values congregate in two categories, those ∼123–166 and those below 100. Another mutation in the latter category besides the *Tmc1* p.W554L/W554L is at a nearby intracellular site *Tmc1* p.D569N/D569N (Beurg et al., 2019) and had 46 ± 18 (*N* = 5) channels. An additional point worth noting from Table 1 is that all dominant mutations, M412K, T416K, and D569N, show a significantly smaller P_OR_ in 0.04 mm Ca^2+^ than the control. This distinction may provide some clue to the origin of dominant and recessive deafness mutations. However, it is important to note that none of the mutations studied abolished Ca^2+^ block of the channel, which is a distinct property independent of Ca^2+^ permeation.

**Table 1.** Ionic properties of the MET channel in P5–P7 apical OHCs from mice with *Tmc1* missense mutations, all of which cause deafness

Apical OHC *Tmc1; Tmc2*^-/-^	D/R	Max MET current (nA)	Single channel conductance (pS)	P_Ca/Cs_	P_OR_ 0.04 mm Ca	I_0.04 Ca_/I_1.5 Ca_	N_MET_
*Tmc1*^+/+^		1.18 ± 0.16 (*N* = 14)	84.8 ± 3.1 (*N* = 5)	4.25 ± 0.35 (*N* = 8)	0.44 ± 0.06 (*N* = 8)	1.54 ± 0.1 (*N* = 8)	166 ± 23 (*N* = 5)
*Tmc1 ^p.^*^M412K/M412K^(*TMC1 p*.M418K)	D	1.14 ± 0.14 (*N* = 10)	86.2 ± 8.2 (*N* = 5)	1.50 ± 0.12*** (*N* = 10)	0.19 ± 0.07*** (*N* = 7)	1.49 ± 0.08 (*N* = 7)	157 ± 20 (*N* = 5)
*Tmc1^p^*^.T416K/T416K^(*TMC1 p*.T422K)	D	1.15 ± 0.10 (*N* = 18)	87.2 ± 4.5 (*N* = 4)	2.80 ± 0.5*** (*N* = 6)	0.22 ± 0.06*** (*N* = 6)	1.60 ± 0.20 (*N* = 6)	157 ± 14 (*N* = 4)
*Tmc1^p^*^.D528N/D528N^	R	0.55 ± 0.05*** (*N* = 11)	53.3 ± 2.9 *** (*N* = 5)	0.57 ± 0.04*** (*N* = 7)	0.39 ± 0.01* (*N* = 4)	1.48 ± 0.19 (*N* = 4)	123 ± 12 (*N* = 5)
*Tmc1^p^*^.W554L/W554L^	R	0.40 ± 0.10*** (*N* = 12)	88.3 ± 4.0 (*N* = 4)	3.59 ± 0.27** (*N* = 8)	0.34 ± 0.02** (*N* = 6)	1.49 ± 0.08 (*N* = 6)	54 ± 13 (*N* = 4)
*Tmc1^p^*^.D569N/D569N^(*TMC1 p*.D572N)	D	0.34 ± 0.13*** (*N* = 11)	87.5 ± 4.5 (*N* = 5)	1.25 ± 0.11*** (*N* = 10)	0.24 ± 0.09*** (*N* = 7)	1.51 ± 0.1 (*N* = 7)	46 ± 18 (*N* = 5)

D/R, mutations are semi-dominant (D) or recessive (R).

P_OR_ is resting open probability in 0.04 mm extracellular Ca^2+^; I increase is current ratio in 0.04 and 1.5 mm Ca^2+^; N_MET_ number of MET channels is ratio of maximum to single-channel current, SD determined by max current. Each value mean ± SD. Mouse mutations (column 1) also show existing homologous human mutations. Human TMC1 numbering, with total 761 aa, differs from neonatal mouse with 757 aa. Significance tests between mutants and control (*Tmc1*+/+) *t* test:

*p < 0.05,

**p < 0.01,

***p < 0.001. Unstarred values were not significantly different from control *Tmc1*+/+, with *p* > 0.2.

## Discussion

**Figure 10.**
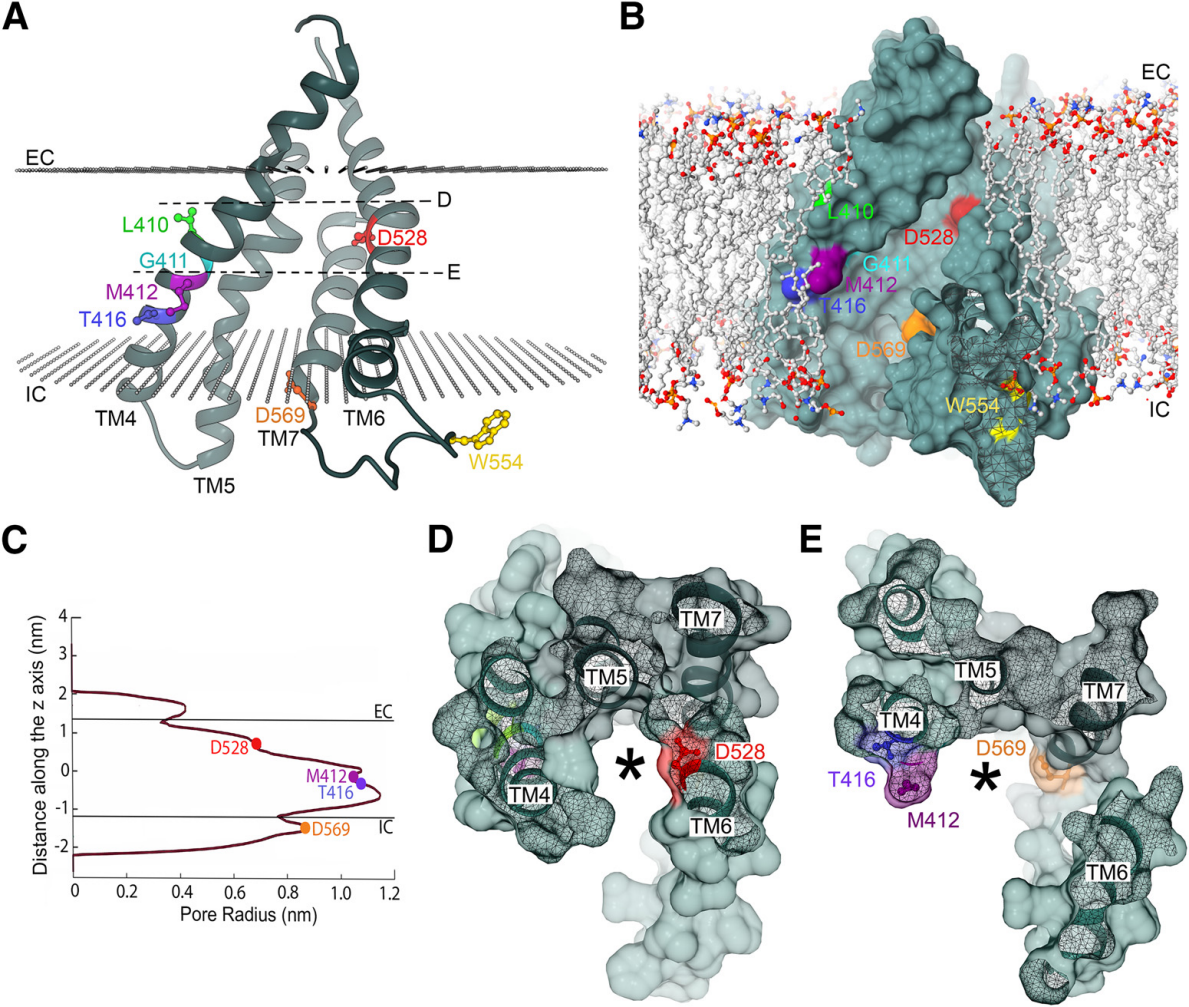
Localization of deafness-causing mutations in TMC1. ***A***, Ribbon representation of the TMC1 cavity built by TM domains TM4–TM7. The side chains of residues L410 (green), G411 (cyan), M412 (magenta), T416 (blue), D528 (red), W554 (yellow), and D569 (orange) are depicted in ball and stick representation. The approximated location of the lipid carbonyls of the plasma membrane is represented with two planes of gray spheres and the intracellular (IC) and extracellular (EC) sides are indicated. D and E dashed lines indicate the planes shown in panels ***D***, ***E***, respectively. ***B***, Surface representation of the TMC1 ion conduction cavity embedded in a lipid membrane. Residues L410, G411, M412, T416, D528, W554, and D569 are colored as in ***A***. Phospholipids are shown in ball and stick representation with their aliphatic chain in gray, phosphate in orange, oxygen in red, and nitrogen in blue. ***C***, Pore profile along the cavity indicating the approximated positions of the EC and IC and residues M412, T416, D528, and D569. ***D***, Surface representation of the top view of the TM4–TM7 cavity from the extracellular side revealing the localization of D528 (red) at its narrower region. ***E***, Surface representation of the top view of the cavity from the extracellular side revealing the localization of D569 (orange), T416 (blue), and M412 (magenta) at the wider region of the cavity. The asterisks in ***D***, ***E*** indicate the potential ion permeation pathway.

We have examined the relation between TMC1 and hair cell transduction by characterizing five missense mutations in the region of the protein, within TM4–TM7, that has been proposed to form the ion conducting pore (Ballesteros et al., 2018; Pan et al., 2018). We described a new dominant human deafness mutation *TMC1* p.T422K and studied the mechanistic consequences in the homologous mouse mutant *Tmc1* p.T416K. We characterized mechanotransduction in this mutant mainly in OHCs at the end of the first postnatal week when there were no noticeable morphologic changes in the hair bundles or cell body. The T416K mutation site is near the *Beethoven* mutation M412K (Vreugde et al., 2002; Marcotti et al., 2006), one turn of the TM4 α-helix closer to the intracellular side of the membrane (Fig. 10*A*,*B*). These two mutations substitute a positively charged for an uncharged residue and have similar effects on the MET channels, including a reduced Ca^2+^ permeability of the channel, a lower resting open probability, P_OR_, in 0.04 mm Ca^2+^ (similar to its concentration in endolymph; Bosher and Warren, 1978) and a smaller susceptibility to block by the aminoglycoside antibiotic DHS. These three properties had been previously documented for the *Beethoven* mutation *Tmc1* p.M412K (Beurg et al., 2015a; Corns et al., 2016). For neither mutant was there an alteration in the single-channel conductance or in the maximum MET current, reflecting an unchanged number of channels in the hair bundle. Besides T416K (human *TMC1* p.G4226R) and the M412K (human *TMC1* p.G418K), there are two other neighboring human deafness mutations, *TMC1* p.G416R and *TMC1* p.L417R, the functional effects of which in mice would be worth studying. Together the effects of these mutations suggest that there is a region of TM4 influencing ion transport. In each case, the deleterious effect of adding a positively charged residue, either lysine (K) or arginine (R), implies an important role of the cavity electrostatics for ion selectivity. The positive charges might depress Ca^2+^ permeation or binding of the cationic DHS. Alternatively, the region of TM4 containing residues 410–416 may constitute the site of interaction of TMC1 with another pore-forming or accessory protein, such as TMIE (Cunningham et al., 2020) or the lipid membrane, and alteration of these interactions could influence the ion selectivity and the open-closed equilibrium.

Only two of the mutations, *Tmc1* p.W554L/W554L and *Tmc1* p.D569N/D569N, showed significantly reduced expression of functional channels in the bundle, N_MET_ (Fig. 8*D*; Table 1). It has been proposed that D569 is the site of interaction of TMC1 with the accessory protein LHFPL5 (Yu et al., 2020) and that the mutation p.D569N impairs this interaction. Since LHFPL5 is required to transport TMC1 into the bundle (Beurg et al., 2015b), reduced interaction between the two proteins is predicted to diminish channel density as observed. Consistent with this notion, we suggest that residue W554 is also within the TMC1-LHFPL5 interaction region. The explanation for the reduced P_Ca_ in *Tmc1* p.W554L/W554L is unclear because the residue is not in the pore cavity according to modeling (Fig. 10*A*,*B*). LHFPL5 may also be a conduit for force to gate the channel since it strongly interacts with the tip link protein PCDH15 (Xiong et al., 2012; Ge et al., 2018). In membrane proteins, tryptophans near the lipid–water interface are thought to interact with the membrane playing an anchoring role (Sanchez et al., 2011; de Jesus and Allen, 2013). The W554L mutation could affect the interaction of the TM6–TM7 loop with the membrane destabilizing this loop and affecting protein stability and folding.

Of the five mutations studied, *Tmc1* p.D528N/D528N has the most severe effects on ion permeation, being the only one to show a reduction (of 37%) in unitary conductance and a large 7-fold reduction in Ca^2+^ permeability; together, these changes are predicted to produce a 10-fold reduction in resting Ca^2+^ influx, more than in any of the other mutations. Furthermore, there was a large, ∼70-fold, reduction in effectiveness of DHS to block the MET channel. According to recent modeling (Ballesteros et al., 2018), the amino acid D528 is near the extracellular side of TM6 in a narrow region of the cavity of a hypothetical channel formed by TMC1 (Fig. 10*B*). The effect on the channel's conductance observed in the D528N mutant could be explained by the removal of a negative charge at this narrow region of the cavity (pore radius of ∼0.65 nm), which would hinder cationic currents (Fig. 10*A–C*). The localization of this aspartate and the severe effects on channel properties implies an important role of this region in ion permeation. In contrast, D569 at the beginning of TM7 and M412 and T416 in the middle of TM4 are facing the wider intracellular region of the cavity with a pore radius of ∼1.1 and 0.9 nm, respectively (Fig. 10*C*). This wider intracellular region would allow ions to permeate more freely or to interact with several water molecules preserving the channel conductance in the p.T416K, p.M412K, and p.D569N mutants. Addition of a positively charged amino acid (M412K or T416K) or the removal of a negatively charged (D569N or D528N) amino acid reduced Ca^2+^ selectivity (Beurg et al., 2019), corroborating the important role of charges along the ion-conducting cavity for preferential selectivity for Ca^2+^ over other cations.

The main aim of our work has been to link mutations causing single amino acid replacements to MET channel structure and performance. The effects of the mutations are consistent with the present view of TMC1 as the primary structural component of the MET channel, with the region of TM4 to TM7 determining its ionic permeability properties (Ballesteros et al., 2018; Pan et al., 2018). The mutations demonstrate that both the Ca^2+^ permeability and unitary conductance can be altered by single amino-acid substitution and they suggest TMC1 binding to at least one other accessory protein LHFPL5. However, an unanswered question is what connects these relatively small alterations in channel properties to subsequent hair cell death and deafness. Clearly all channel variants are manufactured in the endoplasmic reticulum (ER) and transported to the tips of the stereocilia where they behave as functional transducer channels, at least in the first postnatal week. Although for two of the mutants, D569N and W554L, fewer channels are ferried to the transduction site, those that are delivered are fully operative at P6. What then causes the MET channels to lose functionality during subsequent development?

One possible pathway, common to all variants, is a decrease in Ca^2+^ permeability, which in D528N is exacerbated by concomitant reduction in channel conductance, diminishing Ca^2+^ influx into the stereocilia. The reduced Ca^2+^ influx may fail to maintain actin polymerization at the stereociliary tips (Vélez-Ortega et al., 2017) and eventually lead to hair bundle disorganization. In support of this idea, the severity of the mutation, judged by hair cell death at P30, roughly follows the degree of reduction in Ca^2+^ permeability. For example, D528N shows the greatest change in Ca^2+^ selectivity and largest hair cell loss (which includes IHCs) at P30, and W554L the least, with no hair cell loss until P60 (Fig. 6), D569N is intermediate with some IHC loss (Beurg et al., 2019; their Fig. 1*E*). However, this cannot be the whole explanation because all mutants eventually become deaf. A second factor may be the resting open probability in 0.04 mm (endolymph-like) Ca^2+^ which is significantly reduced in the dominant mutations M412K, T416K, and D569N. The main consequence will be a smaller inward current through the open MET channels *in vivo*, causing hyperpolarization of the hair cell resting potential (Johnson et al., 2011). This effect would assume importance in the week after the onset of hearing, P12–P19, when the endolymphatic potential climbs to its maximum of 100 mV (Steel and Barkway, 1989; Li et al., 2020) and as a consequence endolymphatic Ca^2+^ decreases (Johnson et al., 2012). The endolymphatic Ca^2+^ concentration is partly determined by a passive distribution of the ion between the endolymph and perilymph and can be calculated from the Nernst equation: V_EP_ = 30 log_10_ (Ca_P_/Ca_E_), where Ca_P_ and Ca_E_ are the Ca^2+^ concentrations in the perilymph and endolymph, respectively and V_EP_ is the endocochlear potential. Thus, as V_EP_ increases from 0 to 100 mV, the passive component of Ca_E_ should decrease causing an increase in the fraction of MET channels open at rest with non-mutant channels. A third class of mechanism relating the mutations to the hair cell death may be impaired processing of mutant TMC1 in the ER. The reduced Ca^2+^ permeability of the nascent channels may affect ER stress (Krebs et al., 2015) or cytoplasmic Ca^2+^ homeostasis. There is no direct evidence for this last mechanism, but it may influence proper development of the hair cells (Jeng et al., 2020). For example, it is known that in the *Beethoven* mutation, *Tmc1* p.M412K, the voltage-dependent K^+^ currents in both IHCs and OHCs are reduced in amplitude, more so in the homozygote than in the heterozygote (Marcotti et al., 2006). The factors regulating hair cell maturation are still poorly understood, and more experiments are needed to discriminate between the pathways linking MET channel function and hair cell apoptosis.
